# Reactivation of juvenile idiopathic arthritis associated uveitis with posterior segment manifestations following anti-SARS-CoV-2 vaccination

**DOI:** 10.1186/s12348-022-00294-2

**Published:** 2022-04-27

**Authors:** Padmamalini Mahendradas, Sai Bhakti Mishra, Rubble Mangla, Srinivasan Sanjay, Ankush Kawali, Rohit Shetty, Balebail Dharmanand

**Affiliations:** 1 Department of Uveitis and Ocular Immunology, Narayana Nethralaya, Bangalore, India; 2 Department of Cornea and Refractive Surgery, Narayana Nethralaya, Bangalore, India; 3 Department of Rheumatology, Vikram Hospital, Bangalore, India

**Keywords:** Juvenile idiopathic arthritis, JIA associated uveitis, Autoimmune disease, Anti- SARS-CoV-2 vaccination, COVID-19, Fundus fluorescein angiography

## Abstract

**Background/purpose:**

Juvenile idiopathic arthritis (JIA) is the most common rheumatic disease in the pediatric population and anterior uveitis is its commonest extra-articular manifestation. Typically the uveitis presents as chronic anterior uveitis and there is limited literature of the posterior segment manifestations of the disease. Similar to other vaccines, anti-SARS-CoV-2 vaccination that began as an urgent measure to control the spread of the SARS-CoV-2 pandemic has not been without adverse events. We are reporting a 19-year-old Asian Indian female who was diagnosed and treated for JIA associated anterior uveitis that was unilateral and was under anti-inflammatory control but showed worsening of uveitis with posterior segment inflammation in both eyes following anti-SARS-CoV-2 vaccination.

**Case report:**

A 19-year-old Asian Indian female with a history of juvenile idiopathic arthritis on treatment with methotrexate, presented with right eye chronic anterior uveitis with peripheral subclinical retinal vasculitis and macular edema which was brought under control following administration of adalimumab. She was inflammation free for 6 months until she received anti-SARS-CoV-2 vaccination and developed new onset floaters in both eyes that were initially noted after the first dose and increased after the second dose. Clinical examination revealed presence of keratic precipitates and grade 1+ anterior chamber inflammation along with vitiritis in both eyes. Fundus fluorescein angiography revealed angiographically active retinal vasculitis without the presence of macular edema in both eyes. This was managed with a short course of topical difluprednate and continuation of systemic immunosuppressive therapy with adalimumab and methotrexate.

**Conclusion:**

JIA associated uveitis results from an autoimmune process which can be controlled with timely immunosuppressive treatment. It is important to be aware of the potential risk of flare up of uveitis with posterior segment manifestations following anti- SARS-CoV-2 vaccination.

## Introduction

Anti-SARS-CoV-2 vaccination began as an urgent measure to contain the spread of the Coronavirus Disease 2019 (COVID-19) pandemic with massive vaccination drives being conducted all over the world. Similar to previous vaccines, there have been adverse effects, some of which were expected from the earlier phase 1 and 2 trials, while a few others that remain to be noted from the larger post-marketing surveillance phases [1]. Thus, it is critical to report any adverse effects occurring after vaccination for improving our understanding of not only the de novo reactions but also its effect on previously existing diseases such as autoimmune conditions.

Juvenile idiopathic arthritis (JIA) is the most common rheumatic disease in the pediatric population and uveitis is its commonest extra-articular manifestation [2, 3]. While this uveitis has been typically noted to be an asymptomatic chronic anterior uveitis until complications such as band-shaped keratopathy and cataract occur, there is limited literature of the posterior segment manifestations of the disease [4]. We are reporting a young Asian Indian female who was diagnosed and treated for JIA associated uveitis and was under anti-inflammatory control but showed worsening of uveitis with posterior segment manifestations following anti-SARS-CoV-2 vaccination.

## Case report

A 19-year-old Asian Indian female presented to us in December 2020 with complaints of decreased vision in the right eye since 2 weeks. Since the age of 10 years, she had a history of juvenile idiopathic arthritis with ANA positivity by immunofluorescence assay and was on systemic immunosuppressive treatment with methotrexate 20 mg weekly dose at the time of presentation. She also had a history of recurrent anterior uveitis in the right eye since 2007 and had been on and off topical steroids during those flare ups.

Family history revealed HLA-B27 positive ankylosing spondylitis in her father who was on treatment with adalimumab and methotrexate for the same. However, our patient was negative for HLA-B27. She had no history of or any known contact with COVID-19 affected personnel in the recent past.

**Fig. 1:**
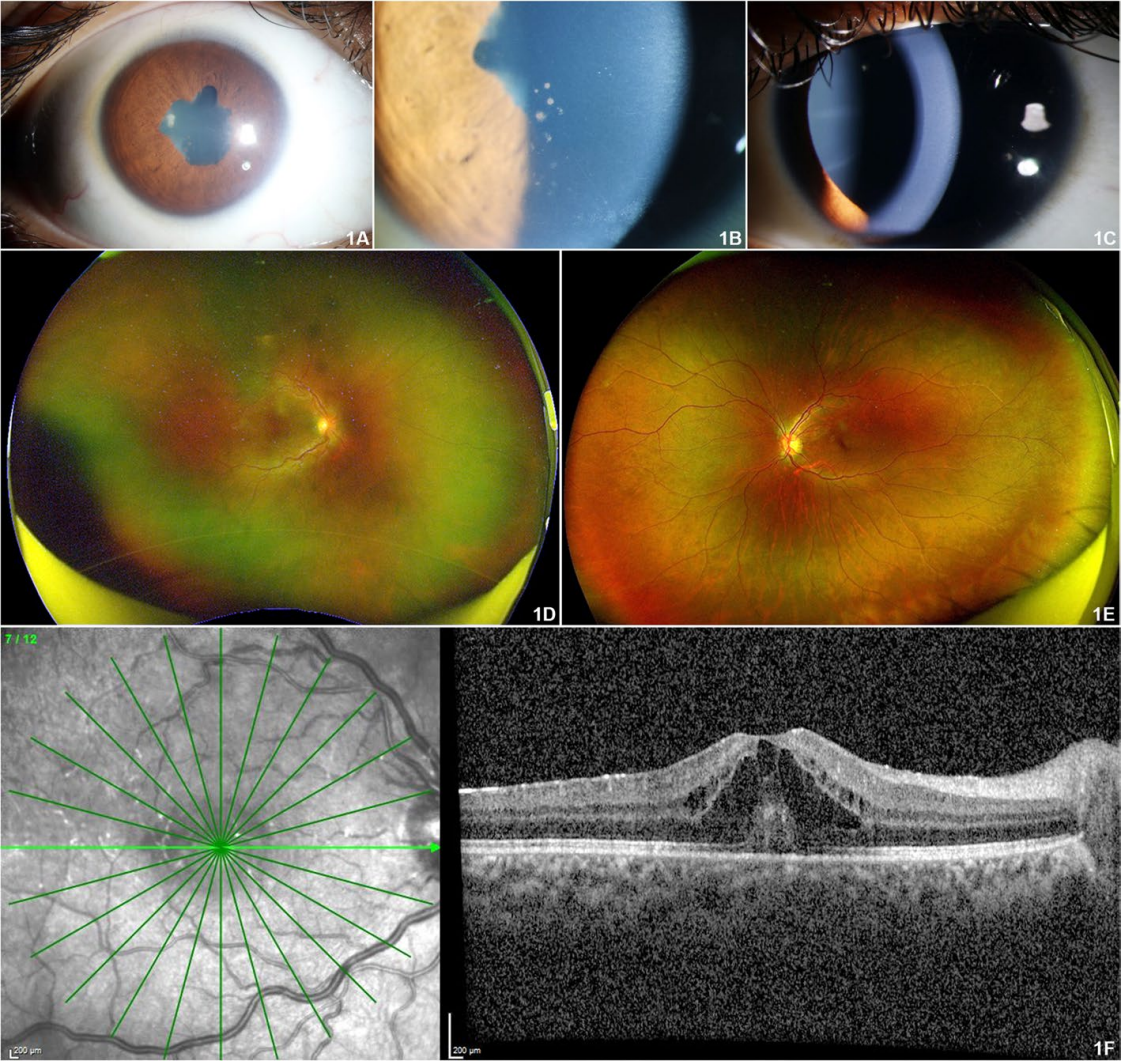
**A** Anterior segment photograph of the right eye showing posterior synechiae and lens changes. **B** Anterior segment broad slit beam photograph of the right eye showing posterior synechiae and pigmented keratic precipitates. **C** Anterior segment photograph of the left eye showing normal anterior segment with pharmacologically dilated pupil. **D** Optos™ wide angle fundus photograph of the right eye revealed grade two media haze, with cystoid macular edema. **E** Optos™ wide angle fundus photograph of the left eye revealed normal posterior segment. **F** Spectral domain optical coherence tomography (SD-OCT) of the right e eye revealed altered foveal contour, multiple intraretinal hyporeflective cystic spaces with high reflective intervening septaes suggestive of cystoid macular edema

On examination, her best corrected visual acuity (BCVA) was 20/60 and 20/20 in the right eye and left eye respectively. Anterior segment examination of the right eye revealed circumciliary congestion, presence of non-granulomatous keratic precipitates, anterior chamber cells 2+ and flare 2+ (SUN grading), 270 degrees posterior synechiae and complicated cataract (Fig. 1A, B). Left eye was quiet on anterior segment evaluation (Fig. 1C). Intraocular pressures were normal in both eyes. Dilated fundus evaluation revealed presence of 1+ vitreous haze with a hyperemic disc and a dull foveal reflex in the right eye (Fig. 1D) with a normal left eye fundus (Fig. 1E).

Optical coherence tomography of the right eye revealed presence of posterior vitreous cells, epiretinal membrane (ERM), cystoid macular edema (CME) with central retinal thickness of 552 μm (Fig. 1F). Left eye macula had a central retinal thickness of 236 μm and appeared normal. Investigations for infectious and non-infectious causes revealed normal blood counts with raised erythrocyte sedimentation rate (ESR 32 mm/hr) and C-reactive protein (CRP 7.69 mg/L). Urine routine and microscopy, liver function and renal tests, vitamin B12 levels, serum ferritin, procalcitonin, D-dimer and lactose dehydrogenase were within normal limits. She was HLA-B27 negative and also seronegative for tuberculosis, syphilis, hepatitis B and C and HIV 1 and 2.

**Fig. 2:**
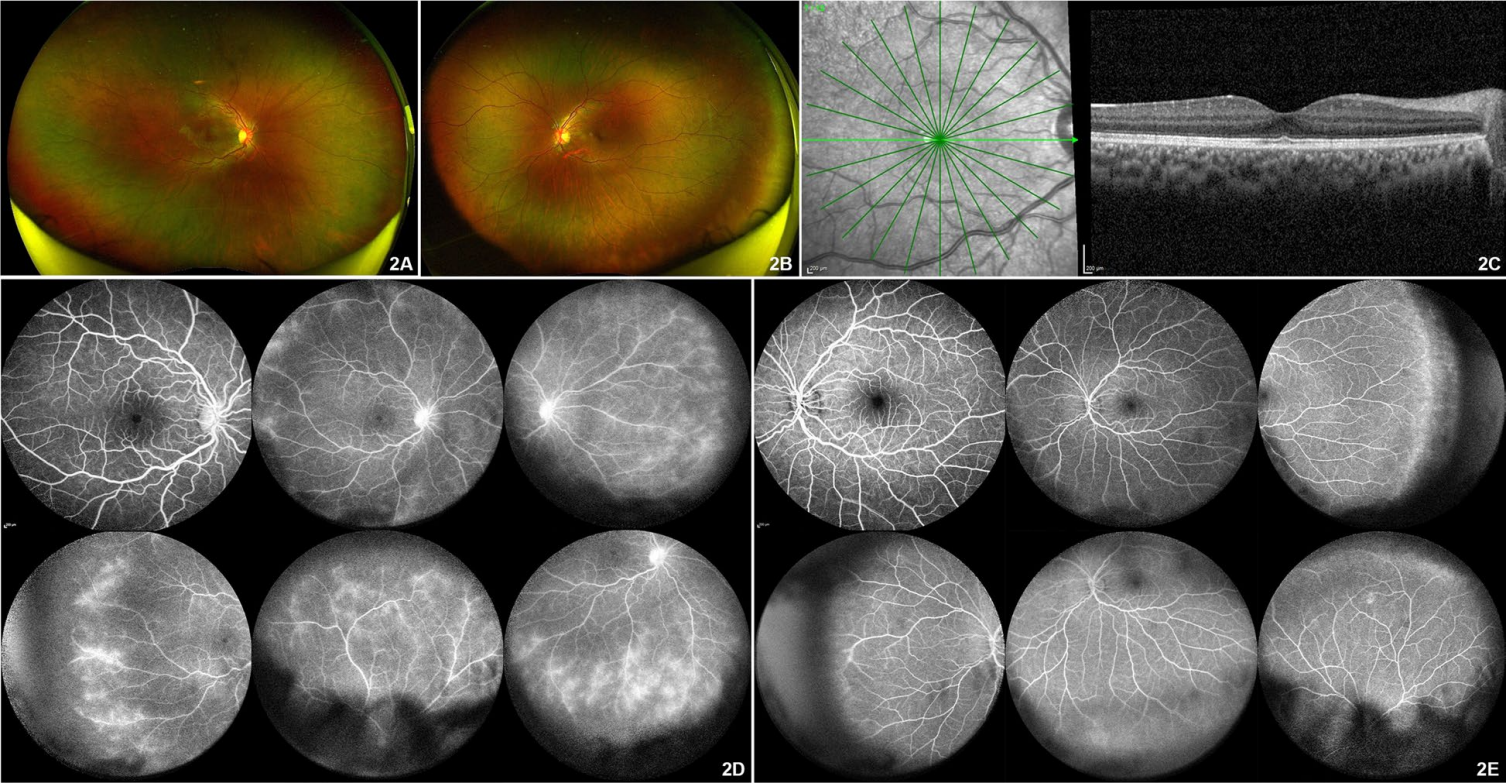
Follow up images after 6 weeks. **A** Optos™ wide angle fundus photograph of the right eye revealed vitreous haze grade one with absent foveal reflex in the right eye. **B** Optos™ wide angle fundus photograph of the left eye was normal. **C** Spectral domain optical coherence tomography (SD-OCT) of the right eye revealed resolved macular edema. **D** Fundus fluorescein angiography (FFA) images of the right eye revealed disc leak, diffuse perivascular leak in the retinal periphery with capillary non perfusion in the temporal retinal periphery. **E** FFA images of the left eye revealed normal angiogram in the posterior pole with minimal perivascular staining in the nasal retinal periphery

She was started on topical steroids (prednisolone acetate 1% six times daily), cycloplegic agent (homatropine hydrobromide 2% twice daily), along with nonsteroidal anti-inflammatory agent (NSAIDS, nepafenac 0.1% thrice daily) and lubricants (carboxymethylcellulose 0.5%) and also continued on methotrexate 20 mg weekly systemic immunosuppressive therapy. Following a rheumatologist consultation, she received subcutaneous injections of adalimumab 40 mg every 2 weeks. At 1 month follow up, she showed signs of improvement with right eye vision improving to 20/40, resolved anterior segment inflammation and macular edema (Fig. 2A). The left eye was normal (Fig. 2B). Fundus fluorescein angiography however showed patchy hypofluorescenece due to blocked fluorescence of vitritis and late hyperfluorescence with late diffuse perivascular leak noted peripherally suggestive of subclinical or angiographically active peripheral retinal vasculitis in the right eye (Fig. 2D). Left eye did not reveal any perivascular leakage (Fig. 2E). There was no macular leakage which corroborated with the resolution of CME with CMT of 305 μm on OCT in the right eye (Fig. 2C). Her methotrexate dose was increased to 25 mg per week and subcutaneous adalimumab 40 mg once in 2 weeks was continued that subsequently led to the resolution of inflammation in the right eye.

After 6 months, she presented with a fresh complaint of floaters associated with mild blurring of vision in both eyes since 1 month. She reported the first appearance of bilateral floaters within a week of the first dose of SARS-CoV-2 (Severe acute respiratory syndrome coronavirus 2) vaccination, Covaxin (Bharat Biotech International Ltd., BBIL). She noticed an increase in the floaters following the second dose that she received after 30 days of the first dose. She presented to us after the second dose of vaccination. She was apparently symptom-free before the vaccination and did not report any symptoms suggestive of systemic infections. She had skipped one dose of methotrexate in the week of vaccination as per advice of the treating rheumatologist.

**Fig. 3:**
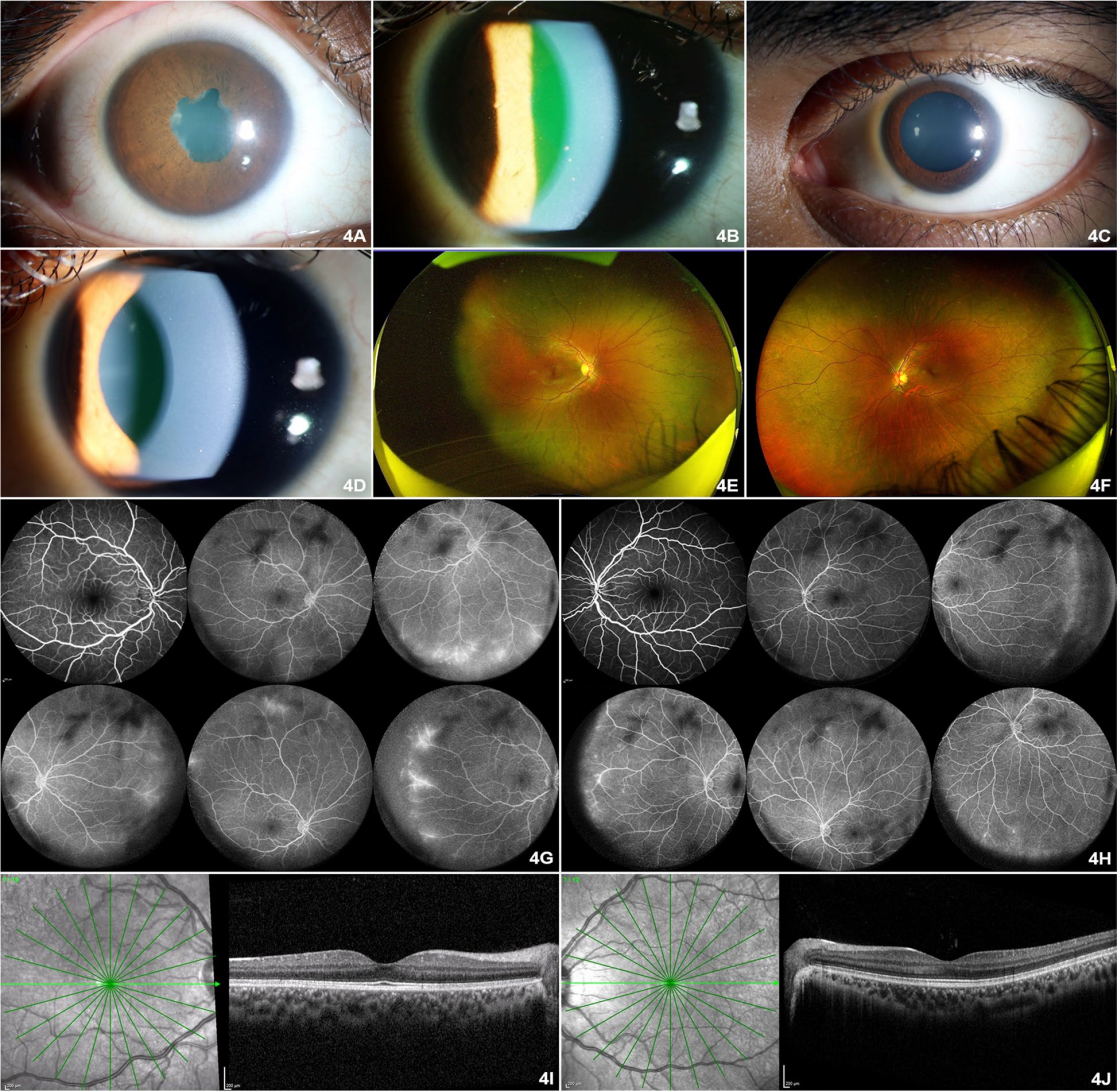
After anti SARS CoV-2 vaccination. **A** Anterior segment photograph of the right eye showing posterior synechiae and lens changes. **B** Anterior segment broad slit beam photograph of the right eye showing multiple pigmented keratic precipitates. **C** Anterior segment photograph of the left eye showing pharmatoclogically dilated pupil. **D** Anterior segment broad slit beam photograph of the left eye showing keratic precipitate. **E** Optos™ wide angle fundus photograph of the right eye revealed grade one media haze, with absent foveal reflex. **F** Optos™ wide angle fundus photograph of the left eye revealed normal posterior segment. **G** FFA images of the right eye revealed blocked fluorescence corresponds to vitreous opacities, disc staining, diffuse perivascular leak in the retinal periphery with capillary non perfusion in the temporal retinal periphery. **H** FFA images of the left eye revealed blocked fluorescence corresponds to vitreous opacities, multiple areas of staining and leakage from the peripheral retinal vessels. **I** SD-OCT of the right eye revealed normal macula in the right eye. **J** SD-OCT of the left eye revealed hyperreflective dots in the posterior vitreous cavity, normal retinal layers with hyperreflective dots in the inner aspect of the choroid

**Fig. 4:**
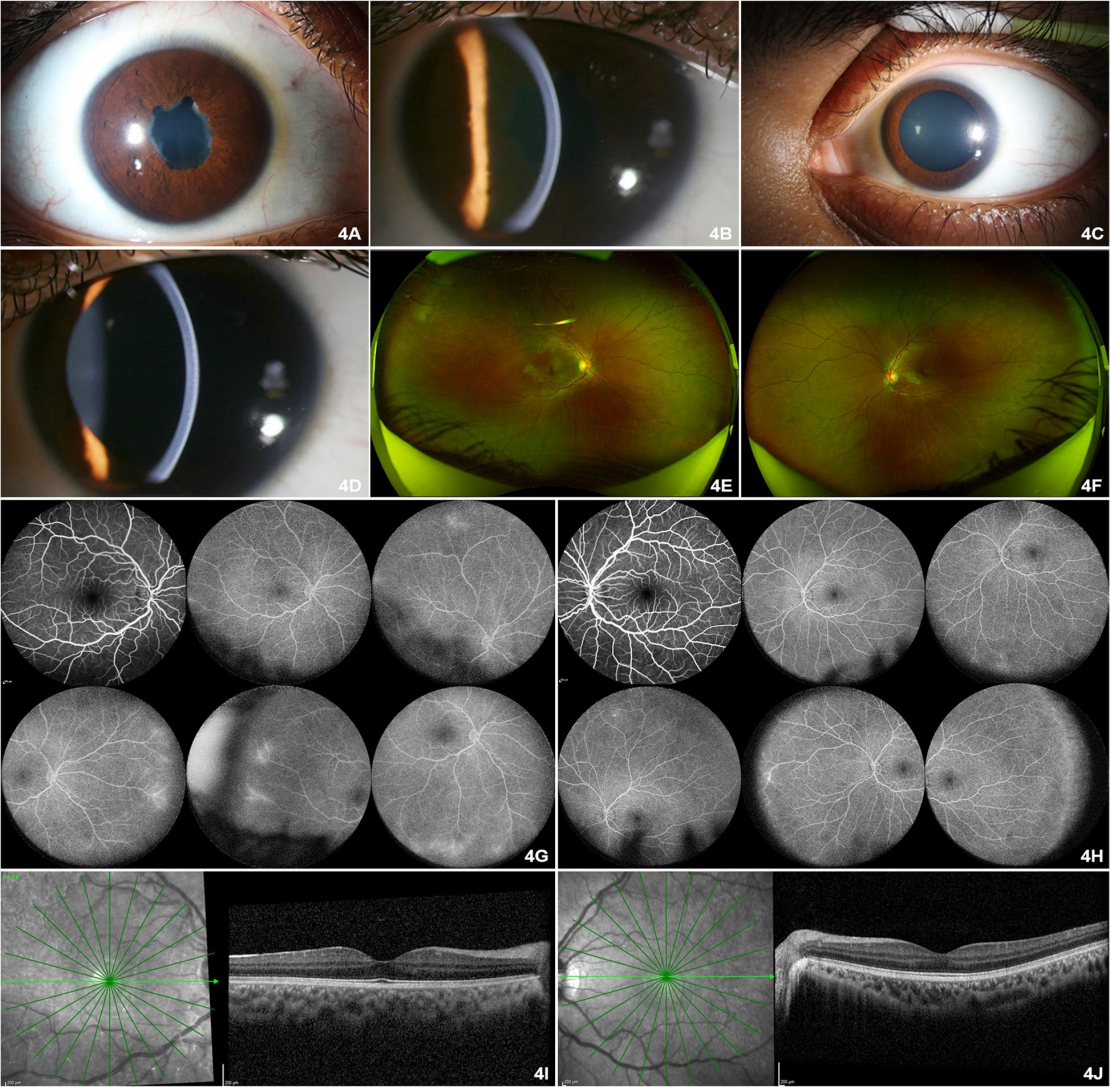
**A** Anterior segment photograph of the right eye showing posterior synechiae and lens changes. **B** Anterior segment slit beam photograph of the right eye showing resolved pigmented keratic precipitates. **C** Anterior segment photograph of the left eye showing pharmacologically dilated pupil. **D** Anterior segment broad slit beam photograph of the left eye showing normal anterior segment structures. **E** Optos™ wide angle fundus photograph of the right eye revealed grade one media haze, with absent foveal reflex. **F** Optos™ wide angle fundus photograph of the left eye revealed normal posterior segment. **G** FFA images of the right eye revealed normal optic disc with reduced perivascular leaks in the retinal periphery with capillary non perfusion in the temporal retinal periphery. **H** FFA images of the left eye revealed reduced areas of staining and leakage from the peripheral retinal vessels. **I** SD-OCT of the right eye revealed normal macula in the right eye. **J** SD-OCT of the left eye revealed normal macula in the left eye

On examination, her BCVA was 20/40 in the right eye and 20/25 in the left eye. Anterior segment examination revealed presence of anterior chamber cells 0.5+ and flare 1+ in both eyes (Fig. 3A-D). There was presence of anterior vitreous cells 2 + with mild disc hyperemia in both eyes (Fig. 3E, F). Fundus fluorescein angiography revealed disc hyperfluorescence with mild leak and diffuse perivascular leak in the peripheral vessels in both eyes (Fig. 3G, H). She was reinvestigated to rule out superadded infectious aetiology and was seronegative for tuberculosis, syphilis and human immunodeficiency virus. Her blood counts, ESR, CRP, liver function tests, renal function tests, urine routine and microscopy were all within normal limits. Patient was treated with topical difluprednate four times a day with gradual taper over 6 weeks with continued bimonthly dose of 40 mg adalimumab and weekly dose of 25 mg methotrexate. Eventually, inflammation resolved on follow up at 6 weeks and visual acuity improved to 20/20 in both eyes (Fig. 4 A–J).

## Discussion

With the global rollout of various anti-SARS-CoV-2 vaccination programmes the world has been able to partially control the spread of the COVID-19 pandemic. Although highly effective and well tolerated, vaccination is not without adverse effects, and the same have been noted with the recent COVID-19 vaccination [5–7]. Our patient developed new onset floaters that were noticed within 1 week following the first dose of vaccination that increased after the second dose, which was when she presented to us. Recurrence of inflammation particularly in the left eye which was uninvolved for past 10 years all of a sudden after vaccination strongly points towards vaccination acting as trigger for this activity, taking into account that other causes of uveitis were ruled out with through investigations and there were no other alterations in her health status, lifestyle or treatment that had changed.

JIA associated uveitis typically presents with a chronic anterior uveitis and very few cases with posterior segment manifestations of the disease have been reported [8–10]. Tripathy K et al. studied the widefield angiography (WFA) features in children less than 16 years with uveitis associated with juvenile idiopathic arthritis and found more than 70% patients showed WFA evidence of posterior segment inflammation [11]. They also found that the decision to change the plan of management was made in 8 of 9 patients who had bilateral quiet anterior chambers after WFA results [11]. This shows the importance of detailed posterior segment evaluation in JIA patients who may have nil to mild anterior segment inflammation as in our patient. Cystoid macular edema which is a common sight threatening complication causing vision loss in these patients, has been attributed to as a complication of the chronic anterior uveitis [12]. In the absence of macular edema, the posterior segment is rarely evaluated in detail, especially using angiography techniques, due to complications such as band-shaped keratopathy and complicated cataract which hinder visualization and examination. The subclinical or angiographically active vasculitis with cystoid macular edema in our patient had resolved with adalimumab and she had obtained inflammatory control of her JIA associated uveitis during her initial visit. At that time, all other causes of posterior uveitis had been ruled out. Six months later, following vaccination when she had a flare up of inflammation, fundus fluorescein angiography revealed posterior segment manifestations with bilateral peripheral retinal vasculitis without macular edema and when she was symptomatic with new onset floaters. Fundus fluorescein angiography is a highly sensitive modality to assess occult reactivation in the form of subclinical vasculitis and should be used in suspected cases for timely increase in immunosuppression to obtain good anti-inflammatory control and visual outcomes.

There could be three alternative possible reasons for such an occurrence of inflammation or increase in ocular inflammation with posterior segment manifestations in our case. Firstly, as Oray M et al. found hypotony and the presence of posterior synechiae at the initial visit as predictive factors of ongoing inflammation into adulthood in patients with JIA-associated uveitis [13]. However, our patient had posterior synechiae in the right eye while the left eye was normal, she never developed hypotony at any time during her 10-year follow up.

Secondly, she was advised to skip one dose of methotrexate in the week of scheduled vaccination. This recommendation followed some studies which suggested that methotrexate treatment may reduce the effectiveness of the vaccines as it causes blunted humoral and cell-mediated immune response in patients with inflammatory diseases [14, 15]. Further, researchers had also suggested strategies such as need for additional vaccine doses or a temporary methotrexate discontinuation that may be required to improve vaccine effectiveness in methotrexate-treated patients [16, 17]. Nevertheless, one skipped dose of methotrexate is unlikely to produce such inflammation in both eyes.

Thirdly, vaccine associated uveitis is also a well-documented phenomenon and has been reported with most vaccines till date [18, 19]. A study reported preponderance of female cases similar to our patient however they found a mean age of 30 years [19]. Proposed hypothesis for vaccine associated uveitis includes both molecular mimicry between vaccine peptides and uveal antigens and antigen specific B cell and T cells induced delayed type of hypersensitivity leading to immune complex deposition causing inflammation [18–20]. Another proposed mechanism is a phenomenon known as Shoenfeld syndrome. It refers to auto-inflammatory and autoimmune conditions that are induced by adjuvants in the vaccine and are likely to occur in patients with a family history of autoimmune disease [21, 22].

Nonetheless, in our case, the possibility of reactivation of juvenile idiopathic arthritis associated uveitis with posterior segment manifestations following anti-SARS-CoV-2 vaccination is most likely. The patient complained of new onset of floaters after the first dose, and worsening of floaters after the second dose. This could be explained by the increased immunogenicity of the second dose. More interestingly, the underlying potential association between flare up of uveitis following both doses of vaccine administration must be considered since it guarantees temporality and consistency of events following vaccination [23].

The causality is also plausible as some biological mechanisms have already been proposed and multiple reports of various other forms of uveitis following vaccination have also been reported. Both infectious and non-infectious diseases have been reported to occur following COVID-19 vaccination [24–33]. Psichogiou et al. reported a series of seven immunocompetent patients of age more than 50 years who developed a reactivation of varicella zoster virus (VZV) following COVID-19 vaccination [25]. We have earlier reported reactivation of VZV causing acute retinal necrosis following COVID-19 vaccination in an elderly Asian male [26].

Albeit rare, associations between vaccination and autoimmunity have been reported in the past [27]. Maillefert et al. reported the development, exacerbation or relapse of rheumatic disorders following hepatitis B vaccination, though a causal relationship could not be established [28]. El Sheikh RH et al. have recently reported new onset acute anterior uveitis following COVID-19 vaccination in a 18 year old girl with a history of ANA positive juvenile idiopathic arthritis [29]. Our patient also developed new onset inflammation in the left eye following vaccination, presenting with bilateral disease for the first time following COVID-19 vaccination. Papasavvas I et al. reported the reactivation of VKH disease that was under control for more than 6 years following covid-19 vaccination [30]. Two cases of allograft rejection following corneal transplantation that occurred after COVID-19 vaccination have been reported by Phylactou et al. Both the patients were treated with topical steroids and showed reversal of rejection [31].

Chau et al. raised doubts regarding antibody dependent enhancements following COVID-19 vaccination that may potentially worsen existing chronic ocular diseases [32]. Rabinovitch T et al. have reported a case of MEWDS in addition to anterior uveitis cases following BNT162b2 mRNA vaccine [33].

Since the administration of COVID-19 vaccinations, numerous reports have discussed different adverse ocular effects following vaccination. In a recent study, Haseeb AA et al. compiled retrospective clinical data of all published reports, within 1 year, of cases with various ocular manifestations following vaccination against COVID-19 [34]. The found a marginal predominance of the female gender (54.9%) among reported cases. AZD1222 ChAdO× 1 nCoV-19, Covishield (Serum Institute of India vaccine, also marketed as (AstraZeneca, Cambridge, UK) was reported 20 (22.9%) times, second only to BNT162b2 mRNA SARS-CoV-2 (BioNTech/Pfizer, Mainz, Germany), which was reported 55 (63.2%) times [34].

With the reporting of similar adverse events we will soon gain a better understanding of the various immunological phenomena precipitating such events and identify vulnerable subpopulations and encourage vigilance regarding symptoms and signs that would require prompt medical care.

## Conclusion

Reactivation of JIA associated uveitis is another potential adverse effect of anti-SARS-CoV-2 vaccination which may manifest with posterior segment manifestations. Prompt medical consultation should be encouraged in patients with symptoms or signs suggestive of flare up of inflammation. However, the medical benefits of covid vaccination far outweigh the risks of flare up of inflammation in this vulnerable group.

## Data Availability

Available on requests.

## Abbreviations

SARS Cov-2: Severe acute respiratory syndrome coronavirus 2
COVID-19: Coronavirus disease 19
JIA: Juvenile idiopathic arthritis
FFA: Fundus fluorescein angiography
VZV: Varicella-zoster virus
BCVA: Best corrected visual acuity
VKH: Vogt-Koyanagi-Harada
ESR: Erythrocyte sedimentation rate
CRP: C-reactive protein
HIV: Human immunodeficiency virus
CME: Cystoid macular edema
ERM: Epiretinal membrane
HLA B27: Human leucocyte antigen B27
ANA: Anti-nuclear antibodies
